# Supplementary material for: Comparative analysis of radiological outcomes among cephalomedullary nails: helical, screw and winged screw
Source: PeerJ. 2024 Sep 19;12:e18020. doi: 10.7717/peerj.18020 (PMC11416753; doi:10.7717/peerj.18020)
Supplement: Supplemental Information 1 [file peerj-12-18020-s001.docx]

STROBE Statement—checklist of items that should be included in reports of observational studies

|  | Item No. | Recommendation | Page  No. | Relevant text from manuscript |
| --- | --- | --- | --- | --- |
| **Title and abstract** | 1 | (*a*) Indicate the study’s design with a commonly used term in the title or the abstract | 1 | Intertrochanteric fractures in patients aged 50 years and older treated with CMN between January 2016 and December 2021 were reviewed retrospectively |
|  |  | (*b*) Provide in the abstract an informative and balanced summary of what was done and what was found | 1 | **Methods:** Intertrochanteric fractures in patients aged 50 years and older treated with CMN between January 2016 and December 2021 were reviewed retrospectively. 158 cases meeting the criteria were included to final analysis. Cases were divided into 3 groups based on the type of nail used (helical blade: group 1, n=54; screw: group 2, n=53 DLT: group 3, n=51). Demographic characteristics, mechanical complications, reduction quality, tip-apex distances (TAD) and Cleveland zones were compared between the groups. Femoral neck shortening, varus collapse, lag sliding, changes in abductor length were compared between study groups. Factors affecting mechanical complications were also analyzed.  **Results:**  Study groups were homogenic in terms of demographic characteristics, fracture type and reduction quality. Regarding mechanical complications, no statistically significant difference was found between groups. All three implants had similar outcomes on femoral neck shortening, varus collapse and lag sliding. Pooled analysis of 158 cases showed that mechanical complications increase as the quality of reduction decreases (p=0.000) same applies when TAD alters from the desired range (p=0.025) and with non-optimally implanted blade according to Cleveland zones (p=0,000). |
| Introduction | | | |  |
| Background/rationale | 2 | Explain the scientific background and rationale for the investigation being reported | 2 | Optimal implant in treatment of intertrochanteric fractures is still matter of debate. Both extra and intramedullary implant types being subject to that debate, cephalomedullary nails had shown to have some advantages over extramedullary implants. Shorter operation time, better pain management, less blood loss, gradual learning curve are being some of them (5). But with mentioned advantages, CMNs are not freed from its disadvantages. After its first clinical uses in 90s, varus collapse, cut-out, z effect and reverse z-effects were faced complications that lead to search for better CMN type.  PFN with helical blade (PFNA) was first to invented after early types of PFNs with two screws. Although there are published studies suggesting helical blade to be superior to screw type implants in terms of complication rate (6) there was still way to go in the search for ideal implant. Dyna-Locking Trochanteric (DLT) Nail is one of later PFN type that has an additional anchor-like mechanism which aims to achieve better grasp of femoral head. On this study we focused on comparing three different types (Helical blade, Screw type blade and DLT type blade) of cephalomedullary nails in terms of mechanical complications, implant failures and radiologic variables (sliding of neck component, changes in abductor arm, vertical femoral neck shortening and varus collapse). |
| Objectives | 3 | State specific objectives, including any prespecified hypotheses | 2 | Optimal implant in treatment of intertrochanteric fractures is still matter of debate. Both extra and intramedullary implant types being subject to that debate, cephalomedullary nails had shown to have some advantages over extramedullary implants. Shorter operation time, better pain management, less blood loss, gradual learning curve are being some of them (5). But with mentioned advantages, CMNs are not freed from its disadvantages. After its first clinical uses in 90s, varus collapse, cut-out, z effect and reverse z-effects were faced complications that lead to search for better CMN type.  PFN with helical blade (PFNA) was first to invented after early types of PFNs with two screws. Although there are published studies suggesting helical blade to be superior to screw type implants in terms of complication rate (6) there was still way to go in the search for ideal implant. Dyna-Locking Trochanteric (DLT) Nail is one of later PFN type that has an additional anchor-like mechanism which aims to achieve better grasp of femoral head. On this study we focused on comparing three different types (Helical blade, Screw type blade and DLT type blade) of cephalomedullary nails in terms of mechanical complications, implant failures and radiologic variables (sliding of neck component, changes in abductor arm, vertical femoral neck shortening and varus collapse). |
| Methods | | | |  |
| Study design | 4 | Present key elements of study design early in the paper | 3 | 3 different types of cephalomedullary nails compared. Group A: PFNA-II helical type blade (Synthes, Oberdoff, Switzerland) Group B: PFN with screw type blade (Zimmer Natural Nail, Warsaw, IN, USA) Group C: Dyna Locking Trochanteric (DLT) Nail (U&I Corporation, Korea). AO/OTA classification system used for classifying fractures as stable/unstable. While 31A1 fractures classified as stable, 31A2 and 31A3 fractures classified as unstable. One of the key elements for homogeneity between groups was achieved reduction quality. Quality of reduction assessed thru scoring system described by Chang et al (7). Considering Garden alignment and fragment displacement, both in AP and lateral view; scoring 0 to 4. Quality of reductions classified as poor, acceptable and excellent.  Tip-apex distance values obtained on first postoperative x-ray through method described by Baumgartner et al.(8). Measured on x-rays proportioned with known diameter of the neck screw/helical blade. Distance between the tip of the screw and apex of femoral measured in both AP and lateral hip x-rays. Values between 15-25 mm considered appropriate.(9)  Values for lag sliding throughout healing process measured with technique described by Watanabe et al (10) .Technique used described in figure 2. The measured values were scaled by comparing with the known diameter of the screw.  To assess femoral neck shortening in modified technique adapted from Zlowodzki was used (11). To perform that, first postoperative anteroposterior xray of the hip outlined, scanned, and then superimposed on most recent follow-up xray with help of graphic software (Rhinoceros 3D, Robert McNeel & Associates, Seattle, WA). Difference between first postop and last follow-up considered. Changes in vertical plane assessed as femoral vertical neck shortening and changes in horizontal plane assessed as abductor arm shortening. Scaling done thru comparing with known diameter of blade.  Changes in neck-shaft angle in follow-up defined as varus collapse. For the values of varus collapse, lag sliding and abductor arm length; Difference between first post-operative and last follow-up x-rays considered. If patients received a secondary operation due to a mechanical complication, their measured variables did not include into comparative analysis. Spatial location of the tip of blade in femoral head was recorded with Cleveland method (12). Cleveland zone for every hip was noted in first postoperative x-ray. Placement of blade classified as optimal, suboptimal, and moderate. Placement in Zone 2,5 and 8 considered as optimal position. Placement of blade in Zones 4,6,7 and 9 considered as suboptimal placements while Zone 1 and 3 considered as moderate placement (13). |
| Setting | 5 | Describe the setting, locations, and relevant dates, including periods of recruitment, exposure, follow-up, and data collection | 3 | Same postoperative weight bearing, and mobilization protocols applied to all patients. Patients allowed and encouraged weight bearing after day 1 alongside with quadriceps strengthening exercises and mechanical DVT prophylaxis. Follow-up visits scheduled at postoperative 2^nd^,6^th,^ and 12^th^ weeks. AP and lateral hip x-rays obtained in every visit. At 12^th^ week follow-up, orthoroentgenogram obtained for accessing length discrepancy and rotational status of lower extremity. Final follow-up was decided as last follow-up x-ray. |
| Participants | 6 | (*a*) *Cohort study*—Give the eligibility criteria, and the sources and methods of selection of participants. Describe methods of follow-up  *Case-control study*—Give the eligibility criteria, and the sources and methods of case ascertainment and control selection. Give the rationale for the choice of cases and controls  *Cross-sectional study*—Give the eligibility criteria, and the sources and methods of selection of participants | 2 | This study conducted in Ege University School of Medicine, Department of Orthopedics. Ethical approval obtained from Ege University Ethics Committee (Num: 21 1T/02). Informed consent from every patient was obtained. Intertrochanteric fractures treated with PFN between January 2016 and December 2021 were reviewed retrospectively thru electronic patient files. Patients with closed intertrochanteric hip fractures, aged 50 years or older, with at least 6 months of follow-up included to final analysis. Cases with pathological fractures, history of ipsilateral hip surgery, immobile patients prior to fracture, and patients with lack of appropriate x-rays were excluded (Figure 1). Total of 158 patients were included in the study. |
|  |  | (*b*) *Cohort study*—For matched studies, give matching criteria and number of exposed and unexposed  *Case-control study*—For matched studies, give matching criteria and the number of controls per case | 2 | This study conducted in Ege University School of Medicine, Department of Orthopedics. Ethical approval obtained from Ege University Ethics Committee (Num: 21 1T/02). Informed consent from every patient was obtained. Intertrochanteric fractures treated with PFN between January 2016 and December 2021 were reviewed retrospectively thru electronic patient files. Patients with closed intertrochanteric hip fractures, aged 50 years or older, with at least 6 months of follow-up included to final analysis. Cases with pathological fractures, history of ipsilateral hip surgery, immobile patients prior to fracture, and patients with lack of appropriate x-rays were excluded (Figure 1). Total of 158 patients were included in the study. |
| Variables | 7 | Clearly define all outcomes, exposures, predictors, potential confounders, and effect modifiers. Give diagnostic criteria, if applicable | 3 | Tip-apex distance values obtained on first postoperative x-ray through method described by Baumgartner et al.(8). Measured on x-rays proportioned with known diameter of the neck screw/helical blade. Distance between the tip of the screw and apex of femoral measured in both AP and lateral hip x-rays. Values between 15-25 mm considered appropriate.(9)  Values for lag sliding throughout healing process measured with technique described by Watanabe et al (10) .Technique used described in figure 2. The measured values were scaled by comparing with the known diameter of the screw.  To assess femoral neck shortening in modified technique adapted from Zlowodzki was used (11). To perform that, first postoperative anteroposterior xray of the hip outlined, scanned, and then superimposed on most recent follow-up xray with help of graphic software (Rhinoceros 3D, Robert McNeel & Associates, Seattle, WA). Difference between first postop and last follow-up considered. Changes in vertical plane assessed as femoral vertical neck shortening and changes in horizontal plane assessed as abductor arm shortening. Scaling done thru comparing with known diameter of blade. |
| Data sources/ measurement | 8* | For each variable of interest, give sources of data and details of methods of assessment (measurement). Describe comparability of assessment methods if there is more than one group | *4* | To assess femoral neck shortening in modified technique adapted from Zlowodzki was used (11). To perform that, first postoperative anteroposterior xray of the hip outlined, scanned, and then superimposed on most recent follow-up xray with help of graphic software (Rhinoceros 3D, Robert McNeel & Associates, Seattle, WA). Difference between first postop and last follow-up considered. Changes in vertical plane assessed as femoral vertical neck shortening and changes in horizontal plane assessed as abductor arm shortening. Scaling done thru comparing with known diameter of blade.  Changes in neck-shaft angle in follow-up defined as varus collapse. For the values of varus collapse, lag sliding and abductor arm length; Difference between first post-operative and last follow-up x-rays considered. If patients received a secondary operation due to a mechanical complication, their measured variables did not include into comparative analysis. Spatial location of the tip of blade in femoral head was recorded with Cleveland method (12). Cleveland zone for every hip was noted in first postoperative x-ray. Placement of blade classified as optimal, suboptimal, and moderate. Placement in Zone 2,5 and 8 considered as optimal position. Placement of blade in Zones 4,6,7 and 9 considered as suboptimal placements while Zone 1 and 3 considered as moderate placement (13). |
| Bias | 9 | Describe any efforts to address potential sources of bias | 3 | The measured values were scaled by comparing with the known diameter of the screw. |
| Study size | 10 | Explain how the study size was arrived at | 2 | This study conducted in Ege University School of Medicine, Department of Orthopedics. Ethical approval obtained from Ege University Ethics Committee (Num: 21 1T/02). Informed consent from every patient was obtained. Intertrochanteric fractures treated with PFN between January 2016 and December 2021 were reviewed retrospectively thru electronic patient files. Patients with closed intertrochanteric hip fractures, aged 50 years or older, with at least 6 months of follow-up included to final analysis. Cases with pathological fractures, history of ipsilateral hip surgery, immobile patients prior to fracture, and patients with lack of appropriate x-rays were excluded (Figure 1). Total of 158 patients were included in the study. |

Continued on next page

| Quantitative variables | 11 | Explain how quantitative variables were handled in the analyses. If applicable, describe which groupings were chosen and why | 3 | The data obtained in the study were put into the database created in the SPSS 22 (IBM Corporation, Armonk, New York, United States) program and statistical analyzes were performed with the same program. Frequencies and percentages of categorical variables, mean values, standard deviation, median values, min and max. values were calculated. The suitability of the continuous variables to the normal distribution was investigated. Non-parametric methods were preferred for the comparison of independent groups. Multiple comparisons of independent groups in continuous variables were made using Kruskal-Wallis method. Cross tables were prepared for categorical variables, distribution differences of groups were tested with Chi-Square method, Type 1 margin of error was determined as α:0.05 in all statistical comparisons. Level of significance was assigned as p<0.05. |
| --- | --- | --- | --- | --- |
| Statistical methods | 12 | (*a*) Describe all statistical methods, including those used to control for confounding | 3 | The data obtained in the study were put into the database created in the SPSS 22 (IBM Corporation, Armonk, New York, United States) program and statistical analyzes were performed with the same program. Frequencies and percentages of categorical variables, mean values, standard deviation, median values, min and max. values were calculated. The suitability of the continuous variables to the normal distribution was investigated. Non-parametric methods were preferred for the comparison of independent groups. Multiple comparisons of independent groups in continuous variables were made using Kruskal-Wallis method. Cross tables were prepared for categorical variables, distribution differences of groups were tested with Chi-Square method, Type 1 margin of error was determined as α:0.05 in all statistical comparisons. Level of significance was assigned as p<0.05. |
|  |  | (*b*) Describe any methods used to examine subgroups and interactions | 3 | The data obtained in the study were put into the database created in the SPSS 22 (IBM Corporation, Armonk, New York, United States) program and statistical analyzes were performed with the same program. Frequencies and percentages of categorical variables, mean values, standard deviation, median values, min and max. values were calculated. The suitability of the continuous variables to the normal distribution was investigated. Non-parametric methods were preferred for the comparison of independent groups. Multiple comparisons of independent groups in continuous variables were made using Kruskal-Wallis method. Cross tables were prepared for categorical variables, distribution differences of groups were tested with Chi-Square method, Type 1 margin of error was determined as α:0.05 in all statistical comparisons. Level of significance was assigned as p<0.05. |
|  |  | (*c*) Explain how missing data were addressed | n/a | n/a |
|  |  | (*d*) *Cohort study*—If applicable, explain how loss to follow-up was addressed  *Case-control study*—If applicable, explain how matching of cases and controls was addressed  *Cross-sectional study*—If applicable, describe analytical methods taking account of sampling strategy | Did not included into final analysis | Did not included into final analysis |
|  |  | (*e*) Describe any sensitivity analyses | n/a | n/a |
| Results | | | | |
| Participants | 13* | (a) Report numbers of individuals at each stage of study—eg numbers potentially eligible, examined for eligibility, confirmed eligible, included in the study, completing follow-up, and analysed | 4 | Mean age was 73,8 (± 10,4) years ranging from 50 to 96. In group 1 (Helical) mean age of 73,1 (±10,4) years ranging from 50 to 89 years. In group 2 (Screw) mean age of 74,1 (±10,2) years ranging from 50 to 96 years. In group 3 (DLT) mean age of 74,3 (±10,9) years ranging from 52 to 94 years. (p = 0.848). Gender distribution in our series was 60 male and 98 female patients. Group I (15M, 39F), group II (20M, 33F), group III (25M, 26F) (p = 0,081). Distribution between groups were homogeneous in terms of stability of fracture according to AO/OTA classficiation (p=0.196). Mean follow-up period of group 1, 2 and 3 were 19,02 ± 14,04, 18,14 ± 12,94 and 16,19 ± 8,79 respectively. There were no statistically significant differences between the study groups (p = 0,568) (Table 1). |
|  |  | (b) Give reasons for non-participation at each stage | n/a | n/a |
|  |  | (c) Consider use of a flow diagram | Yes | Figure 1 |
| Descriptive data | 14* | (a) Give characteristics of study participants (eg demographic, clinical, social) and information on exposures and potential confounders | 4 | Reduction quality was strongly related with complication rates (p=0,000). Tip-apex distance values were similar in all groups (p=0,443). TAD values classified in 2 groups as appropriate or inappropriate. Appropriate TAD values were associated with lower complication rate while inappropriate values of TAD were associated with higher complication rates (p=0,025). Moderate and suboptimal implanted blades were significantly related with mechanical complications (p=0,000). |
|  |  | (b) Indicate number of participants with missing data for each variable of interest | n/a | n/a |
|  |  | (c) *Cohort study*—Summarise follow-up time (eg, average and total amount) | 4 | Mean follow-up period of group 1, 2 and 3 were 19,02 ± 14,04, 18,14 ± 12,94 and 16,19 ± 8,79 respectively. There were no statistically significant differences between the study groups (p = 0,568) (Table 1). |
| Outcome data | 15* | *Cohort study*—Report numbers of outcome events or summary measures over time | *4* | Total of 29 patients faced mechanical complications (Table 3). Cut-out was most common (n=17, 10.7%) mechanical complication that followed by peri-implant fracture (n=4, 2.5%). Other complications were non-union (n=4, 2.5%) implant failure n=4, 1.2%). and avascular necrosis (n=1, 0.6%). Comparing mechanical complication rates, no statistically significant difference was found between three groups. Pooled analysis of 158 cases, showed that mechanical complications increased as the reduction quality decreased (p=0,000), when TAD is not in the desired range. (p=0,025) and when blade is not implanted optimally according to Cleveland index (p=0,000). |
|  |  | *Case-control study—*Report numbers in each exposure category, or summary measures of exposure |  |  |
|  |  | *Cross-sectional study—*Report numbers of outcome events or summary measures |  |  |
| Main results | 16 | (*a*) Give unadjusted estimates and, if applicable, confounder-adjusted estimates and their precision (eg, 95% confidence interval). Make clear which confounders were adjusted for and why they were included | n/a | n/a |
|  |  | (*b*) Report category boundaries when continuous variables were categorized | 4 | Complication rate for our series was 18,4%. In group 1 (Helical) it was 24,1%, in group 2 (Screw) it was 17,0% and in group 3 (DLT) it was 13,7%. Complication rate between study groups were statistically insignificant. Total of 4 peri implant fractures were seen in our series and all 4 of them were in group A but there this variable was not statistically significant.  Evaluating varus collapse in each group, there were no statistically significance difference between groups (p=0,954). All 3 implants managed to prevent varus collapse effectively. For the vertical shortening of femoral head calculations again there were no significant differences while there were slightly less shortening in group A (2,00 vs 3,35 and 4, 80 respectively) (p=0,343). Horizontal neck shortening which can be interpreted as abductor arm shortening were similar in all groups while in group C there were less shortening (0,35 vs 2,10 and 3,30) (p=0,115). Lag sliding measurement were similar between all three groups while in group C median values were higher, but this was not statistically significant (Table 4 |
|  |  | (*c*) If relevant, consider translating estimates of relative risk into absolute risk for a meaningful time period |  |  |

Continued on next page

| Other analyses | 17 | Report other analyses done—eg analyses of subgroups and interactions, and sensitivity analyses | n/a | n/a |
| --- | --- | --- | --- | --- |
| Discussion | | | | |
| Key results | 18 | Summarise key results with reference to study objectives | 4,5 | Using intramedullary nailing techniques, fractures heal with secondary bone healing. In these techniques, gains achieved by the minimally invasive surgeries are countered by relatively bigger gap at the fracture line and lesser stability of fracture than absolute fixation (14). Intraoperative compression acquired thru implants aims to minimize this risk by reducing the fracture gap. Too big of a gap or continuous motion thru sliding on a fracture line could disrupt bone healing. Regarding individualistic nature of healing, defining “healing” on a scale that could enclose all variables is a challenging task. However, reoperation rate suggested as a good tool for this purpose (15). Our results, comparing mentioned device types, are suggesting no difference in reoperation rate between groups. This can be interpreted as all 3 devices were similar regarding final healing status and healing rate. |
| Limitations | 19 | Discuss limitations of the study, taking into account sources of potential bias or imprecision. Discuss both direction and magnitude of any potential bias | 4,5 | Hip fractures in elderly population is challenging in many ways. Comorbidities, bone quality, type of fracture, pre-injury activity level etc. are being factors that we cannot change. Besides, choosing optimal implant, obtaining good reduction quality and appropriate TAD, appropriate postoperative rehabilitation programs are in our hands. Institutional and local facilities and variable access to implants could also lead implant selection ahead of surgeon’s choice. Within the scope of variables evaluated in this study, our results failed to suggest any mechanical/radiological superiority to highlight one device on another. |
| Interpretation | 20 | Give a cautious overall interpretation of results considering objectives, limitations, multiplicity of analyses, results from similar studies, and other relevant evidence | 6 | Limitations for this study were its retrospective nature and limited number of patients because of high rate of loss on follow-ups. Lack of clinical results could be considered as other weakness. Published papers on this population reports fewer follow-up loss. We think reason for that, patients preferred to continue their follow-ups in local/smaller centers. Relatively short mean follow-up period could be considered as another weakness but considering most of mechanical complications occur during first 3 months of follow-up, mean value of 12,7 months can be considered acceptable in aspect of this study. Higher complication rate compared to published papers could be result that patients who developed complications were followed up at a higher rate. |
| Generalisability | 21 | Discuss the generalisability (external validity) of the study results | 6 | A RCT from Korea by Shin et al. with 353 patients, compared ZNN and PFNA. Primary objective was to compare functional outcomes, but reoperation rate and cut-out incidence were also analyzed. While clinical comparison failed to suggest any significant difference between two devices, data on complications was compatible with our study, suggesting implant type alone is not a significant variable for cut-out. Univariate analysis suggested that TAD was the major predictor for cut-out. Reduction quality was not related to cut-out rate (25). There were limited number of published studies about wedge-wing type DLT nail in published literature, and none of them were designed as a comparative study. Retrospectively conducted case series by Temiz et al., Baik et al., and Gunay et al. reported satisfactory clinical and radiologic results with limitations of their designs: they all were one center studies with limited number of patients (26–28). To best to our knowledge this study is first to compare wedge-wing design device to other commonly used cephalomedullary nails in settings of mechanical complications. Possible future reviews with more reported series could suggest higher level data. |
| Other information | |  | | |
| Funding | 22 | Give the source of funding and the role of the funders for the present study and, if applicable, for the original study on which the present article is based | 6 | This study conducted in Ege University School of Medicine, Department of Orthopedics. Ethical approval obtained from Ethics Committee (Num: 21 1T/02). Informed consent from every patient was obtained. Authors declares no conflicts of interest to this manuscript. No fund received for this study. There is no relation with third parties commercially. There is no benefit received in any terms. Dataset for this study could be provided upon request from corresponding author. |

*Give information separately for cases and controls in case-control studies and, if applicable, for exposed and unexposed groups in cohort and cross-sectional studies.

**Note:** An Explanation and Elaboration article discusses each checklist item and gives methodological background and published examples of transparent reporting. The STROBE checklist is best used in conjunction with this article (freely available on the Web sites of PLoS Medicine at http://www.plosmedicine.org/, Annals of Internal Medicine at http://www.annals.org/, and Epidemiology at http://www.epidem.com/). Information on the STROBE Initiative is available at www.strobe-statement.org.
